# Testing the Neuroprotective Properties of PCSO-524^®^ Using a Neuronal Cell Cycle Suppression Assay

**DOI:** 10.3390/md17020079

**Published:** 2019-01-24

**Authors:** Beika Zhu, Yang Zhang, Karl Herrup

**Affiliations:** Division of Life Science, Hong Kong University of Science and Technology, Clear Water Bay, Kowloon, Hong Kong, China; bzhuab@connect.ust.hk (B.Z.); yzhangbs@connect.ust.hk (Y.Z.)

**Keywords:** PCSO-524^®^, neuroinflammation, neuronal cell cycle event, neuroprotection, Alzheimer’s disease

## Abstract

Cell cycle reentry is a unified mechanism shared by several neurodegenerative diseases, including Alzheimer’s disease (AD) and Ataxia Telangiectasia (A-T). This phenotype is often related to neuroinflammation in the central nervous system. To mimic brain inflammation in vitro, we adopted the previously established method of using conditioned medium collected from activated THP-1 cells and applied it to both differentiated HT22 cells and primary neurons. Unscheduled cell cycle events were observed in both systems, indicating the potential of this approach as an in vitro model of neurodegenerative disease. We used this assay to measure the neuroprotective effects of New Zealand green-lipped mussel extract, PCSO-524^®^, to protect post-mitotic cells from cell cycle reentry. We found that, both in vitro and in an animal model, PCSO-524^®^ displayed promising neuroprotective effects, and thus has potential to postpone or prevent the onset of neurodegenerative disease.

## 1. Introduction

The neurons of the mammalian central nervous system (CNS) are fully differentiated cells that are permanently post-mitotic in the adult. During development, as they begin the process of cytological maturation, they permanently lose their ability to replicate their genome and divide into two daughter cells. This life-long prohibition against cell division can be seen most dramatically in a variety of neurodegenerative diseases, including Alzheimer’s disease [1,2,3], ataxia telangiectasia [4], Parkinson’s disease [5], and others. These neurodegenerative conditions differ dramatically in their age of onset and the anatomy of the most prominent neuronal cell losses. Yet in each of them, in the neuronal populations that are at risk for death, significant numbers of post-mitotic neurons show evidence of attempting to reenter a cell cycle [2,6,7,8,9,10].

The molecular mechanisms that link unscheduled neuronal cell cycle to cell death are largely unknown. Despite this, because the linkage is robust and found in several different conditions with neurodegeneration [11,12,13], we have adopted a therapeutic approach based on the assumption that any compound that could interrupt a neuron’s reentrance into a cell cycle process would have potential neuroprotective properties. We are particularly keen to apply this strategy to Alzheimer’s disease (AD), where multiple studies have pointed to brain inflammation as one possible trigger of neuronal cell death [14,15] and ectopic neuronal cell cycle induction [16,17]. Several non-neuronal brain cells are involved in this process, including microglia, the macrophages of the brain, which detect signs of homeostatic disturbance [18] and mount an innate immune response similar to that of a macrophage [18]. Microglia sense structural damage and initiate the local production of cytokines and chemokines, which will in turn activate the immune system [19]. In AD, the presence of aggregated forms of the beta-amyloid peptide (Aβ) are believed to stimulate microglia to release neurotoxic cytokines and chemokines [20,21,22,23,24]. It has been reported that the conditioned medium from Aβ activated human monocyte THP-1 cells and primary microglia could induce neuronal cell cycle and cell death [22]. This observation was later confirmed by other studies that this neuronal cell cycle-related cell death resulted from the pro-inflammatory cytokines TNFα [16] and glutamate [24] secreted by microglia. Based on these studies, we have now adopted an in vitro model of unscheduled cell cycle events triggered by neuroinflammation as a screening technique [22] and used it to screen novel sources of natural products for neuroprotective agents.

In the current work, we sought to apply our strategy to a product that has shown promise as an anti-inflammatory nutritional supplement. Multiple lines of evidence have reported that polyunsaturated fatty acids, such as omega-3 fatty acids, display anti-inflammatory effects in aging and in mouse models of AD [25,26,27,28]. PCSO-524^®^, an extract of the New Zealand green-lipped mussel (Perna canaliculus), is rich in polyunsaturated fatty acids and has strong anti-inflammatory properties [29,30] and, therefore, presented itself as a strong candidate for further investigation. It has received interest as a treatment for arthritis [31,32], but has never been tested for use with neurological conditions of aging, particularly AD.

Taking the advantage of the existing model of unscheduled neuronal cell cycle events, we documented the neuroprotective effect of PCSO-524^®^. We then extended our findings to an animal model of neurodegeneration, where PCSO-524^®^ also proved effective, implying its potential to delay or prevent the onset of neurodegenerative disease.

## 2. Results

### 2.1. Conditioned Medium Induces Differentiated HT22 Cell Cycle Reentry

**Figure 1:**
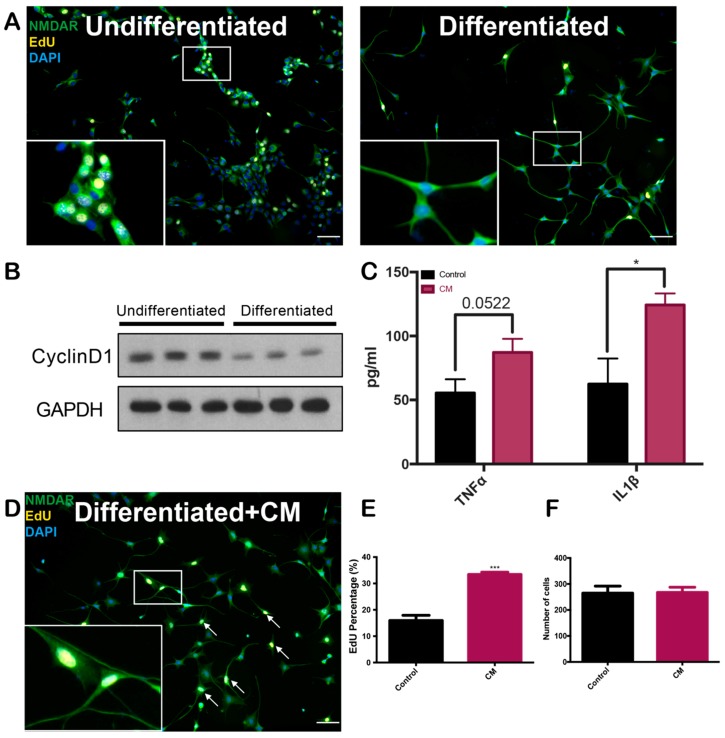
Beta-amyloid stimulated THP-1 cell conditioned medium was mitogenic to differentiated HT22 cells. (**A**) HT22 cells before and after differentiation with dibutyryl cAMP (dbcAMP). Anti-NMDAR antibody (green) was used to reveal the changed morphology of the cells. EdU (yellow) was taken up by cells in the cell cycle and engaged in DNA synthesis. Scale bars, 50 µm. (**B**) Western blot analysis of the G1 phase cell cycle marker, cyclin D1, confirmed that differentiated HT22 cells exited cell cycle; (**C**) ELISA of THP-1 conditioned medium (CM). With fibrillar Aβ stimulation for 72 h, THP-1 cells released pro-inflammatory cytokines, TNFα and IL1β, into their medium. * *p* < 0.05; unpaired Student’s T-test; *n* = 3. Data are means ± SEM. (**D–F**) CM-induced differentiated HT22 cells to reenter a cell cycle. In these experiments, 25% CM was applied to differentiated HT22 cells for 24 h. EdU incorporation increased after CM treatment (**E**), but there was no significant change in cell number (**F**). Arrows point to the EdU-positive differentiated HT22 cells. *** *p* < 0.001; unpaired Student’s T-test; *n* = 3. Scale bars, 50 µm. Data are means ± SEM.

As an initial screen for the effects of the drug on neuronal cell cycle events, we used HT22 cells, a neuroblastoma line that expresses neuron-like characteristics after differentiation [33]. HT22 cells were seeded in 24-well plates and then induced to differentiate by adding 1 mM dibutyryl cyclic AMP (dbcAMP) to their medium. This treatment caused the cells to develop thin neuronal-like processes and exit from the cell cycle [34]. After six days, the cultures were stable in terms of both cell number and cell morphology (Figure 1A). After differentiation, both 5-ethynyl-2′-deoxyuridine (EdU) incorporation (Figure 1A) and the levels of cyclin D1, a G1-phase cell cycle marker [35] decreased (Figure 1B), indicating that the cells had exited the cell cycle. Although not a perfect replicate, differentiated HT22 cells thus represent a satisfactory initial screening platform.

We mimicked the chronic inflammation of the Alzheimer’s disease brain in cell culture, by using human THP-1 monocyte cells [22]. After stimulation with Aβ, THP-1 cells closely mimic the response of primary microglia [20,36,37]. We then dried suspensions of fibrillarized Aβ on the surface of culture dishes to mimic an Aβ plaque in vitro, as we reported previously [22]. Exposed to such Aβ-coated plates, THP-1 cells secrete factors into the medium that are harmful to neurons [22,38]. As confirmation, we collected the conditioned medium (CM) from THP-1 cultures after Aβ stimulation, and used enzyme-linked immunosorbent assay (ELISA) to measure the concentration of the pro-inflammatory cytokines, TNFα, and IL1β in CM. In comparison, we collected the medium from untreated THP-1 cell as a control. The levels of both cytokines were increased above those found in the medium from unstimulated control cultures, suggesting the inflammatory effect of the conditioned medium (Figure 1C).

To investigate whether CM could induce differentiated HT22 cells to re-enter a cell cycle, we replaced 25% of the culture medium of differentiated HT22 cells with CM for 24 h. Compared with the untreated control, there was a two-fold increase in the percentage of EdU-positive cells (Figure 1D,E). Despite this increased cell cycle activity, the number of cells did not decrease significantly after CM treatment (Figure 1F). Of note is the fact that there was also no increase in 4′,6-diamidino-2-phenylindole (DAPI) counts, suggesting that the enhanced EdU uptake was not due to a small portion of cells returning to a normal cell division program. Taken together, the data support the idea that Aβ stimulated THP-1 conditioned medium contains substances that drive differentiated HT22 cells into a cell cycle in a fashion similar to primary cortical neurons [22].

### 2.2. PCSO-524^®^ Protects Against CM-Induced Cell Cycle Reentry

**Figure 2:**
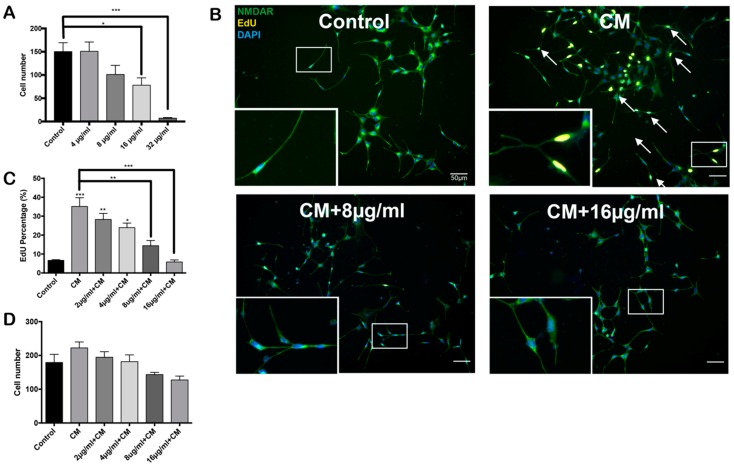
PCSO-524^®^ protects differentiated HT22 cells from CM. (**A**) Toxicity test of PCSO-524^®.^ Differentiated HT22 cells were exposed to different concentrations of PCSO-524^®^ for 24 h. * *p* < 0.05, *** *p* < 0.001; one-way ANOVA with Dunnett’s multiple-comparison test; *n* = 3. Data are means ± SEM. (**B–D**) PCSO-524^®^ pretreatment blocks CM-induced cell cycle activity. Differentiated HT22 cells were treated with indicated concentrations of PCSO-524^®^ for 2 h before the addition of CM. * *p* < 0.05, ** *p* < 0.05, *** *p* < 0.001; one-way ANOVA with Dunnett’s multiple-comparison test; *n* = 3. Although a slight trend was observed, there was no significant change in cell number (**D**). Scale bars, 50 µm. Data are means ± SEM.

**Figure 3:**
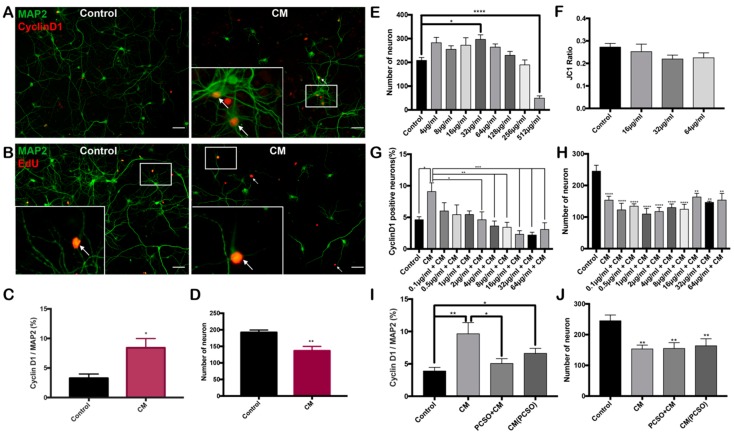
PCSO-524^®^ protects mature neurons from cell cycle reentry. (**A–D**) CM-induced cell cycle events and cell death in mature neurons. Primary neurons were exposed to 6.25% CM for 24 h, leading to a significant increase in cyclin D1 (**A**,**C**), EdU (**B**), and cell loss (**D**). * *p* < 0.05, ** *p* < 0.01; unpaired Student’s T-test; *n* = 6. Data are means ± SEM. Scale bars = 50 µm. (**E–F**) Toxicity test of PCSO-524^®^ on primary neurons. (**E**) Primary neurons were incubated with PCSO-524^®^ for 24 h. Neuron number (MAP2-positive cells declined at high concentrations). (**F**) JC1 dye was added to the neuronal culture to measure mitochondrial integrity. The ratio fluorescence intensity of JC1 monomer and aggregates declined as mitochondrial membrane potential was lost. * *p* < 0.05, **** *p* < 0.0001; one-way ANOVA with Dunnett’s multiple-comparison test; *n* = 3. Data are means ± SEM. (**G–H**) Dose-response curve of the ability of PCSO-524^®^ to block the effects of CM on primary neurons. PCSO-524^®^ was added to cultures of primary neurons 2 h before the addition of 6.25% CM. Neuronal cell cycle events were reduced (**G**), but the loss of MAP2-positive neurons was not (**H**). * *p* < 0.05, ** *p* < 0.01, *** *p* < 0.001, **** *p* < 0.0001; one-way ANOVA with Dunnett’s multiple-comparison test; *n* = 3. Data are means ± SEM. (**I–J**) Pretreatment of THP-1 cells with 16 µg/ml PCSO-524^®^ 2 h before the addition of CM (PCSO) was not protective against the induction of neuronal cell cycle events (**I**) nor cell number loss (**J**). * *p* < 0.05, ** *p* < 0.01; one-way ANOVA with Dunnett’s multiple-comparison test; *n* = 3. Data are means ± SEM.

PCSO-524^®^, an extract from the New Zealand green-lipped mussel, has been demonstrated to exert an anti-inflammatory effect [29]. Before testing its effect on the cell cycle, we performed a toxicity test on differentiated HT22 cells (Figure 2) and neurons (Figure 3). PCSO-524^®^ showed no toxicity on HT22 cells at concentrations below 8 µg/mL. Above this value, however, it caused a significant reduction in HT22 cell number. Next, we asked whether PCSO-524^®^ could protect against the effects of CM. We pretreated differentiated HT22 cells with different concentrations of PCSO-524^®^ for 2 h before the addition of CM, then incubated the cells for another 24 h. By both morphology (Figure 2B) and the percentage of cycling cells (Figure 2C), PCSO-524^®^ significantly blunted the impact of CM (Figure 2C). Although 16 µg/mL PCSO-524^®^ treatment induced a significant cell loss (Figure 2A), its potential in protecting against cell cycle reentry could not be ignored.

### 2.3. PCSO-524^®^ Shows Neuroprotective Effect on Post-mitotic Neurons

With these promising results on differentiated HT22 cells, we next asked if the effect of PCSO-524^®^ was reproducible on primary neurons. We added CM at a final concentration of 6.25% to mature primary cortical neurons for 24 h. This treatment induced a fraction of the primary neurons in our cultures to enter the cell cycle as shown by increased immunostaining for the G1 phase cell cycle marker, cyclin D1. (Figure 3A–C). As predicted, associated with the increased neuronal cell cycle activity, CM also induced significant nerve cell death (Figure 3D), validating our earlier findings that CM-induced post-mitotic neurons to initiate a lethal cell cycle [22]. We used EdU as a second measure of cell cycle reentry. As with the cyclin D1 cell cycle marker, the percentage of MAP2/EdU double-positive cells also increased (Figure 3B). We noticed that with both cyclin D1 and EdU, the neurons identified as cycling were smaller, with short processes and weak MAP2 staining (Figure 3B). This appearance might suggest that, as cells initiated the cell cycle program and transition towards cell death, they shrank in size and reduced their dendritic complexity.

We next asked whether PCSO-524^®^ was toxic to primary neurons. After treating cultures for 24 h, we found that low concentrations of PCSO-524^®^ actually slightly improved neuronal health as measured by increased numbers of MAP2-positive cells in the culture (Figure 3E). This effect reached significance at 32 µg/mL. At concentrations above 64 µg/ml, however, PCSO-524^®^ gradually induced neuronal loss, although this effect did not achieve significance until very high concentrations (512 µg/mL—Figure 3E). These dose-response curves suggest that primary neurons are more resistant to the toxic effects of high concentrations of PCSO-524^®^ than HT22 cells (compare Figure 3E and Figure 2A). To determine whether the lower concentrations of PCSO-524^®^ had cryptic effects that were not captured by a simple measure such as total cell number, we asked whether there was detectable stress on the mitochondrial membrane potential [39]. We used JC1 dye to measure mitochondrial membrane potential [40] and found that below 64 µg/ml, PCSO-524^®^ had virtually no effect on the mitochondrial membrane potential (Figure 3F), indicating that at these lower concentrations it is non-toxic to neurons.

**Figure 4:**
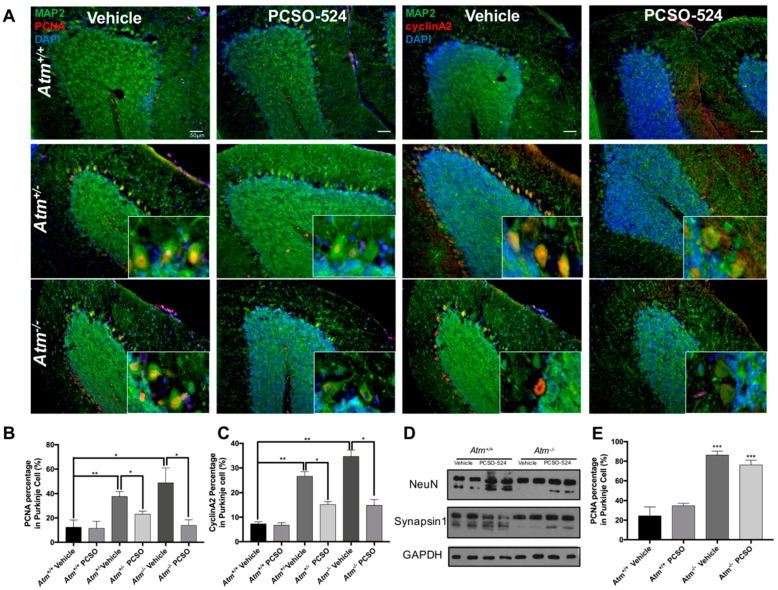
PCSO-524^®^ prevented neuronal cell cycle events and neurodegeneration in vivo. (**A–D**) PCSO-524^®^ was dissolved in olive oil and fed to *Atm*^−/−^ mice daily (25 mg/kg) from P10 to P30. (**A,B,C**) Treatment resulted in a significant decrease in neuronal cell cycle events in Purkinje cells. (**D**) In cerebral cortex, PCSO-524^®^ prevented the loss of the neuronal nuclear protein NeuN and the synaptic vesicle protein, synapsin-I. * *p* < 0.05, ** *p* < 0.01; one-way ANOVA with Dunnett’s multiple-comparison test; *n* = 6. Scale bars, 50 µm. Data are means ± SEM. (**E**) PCSO-524^®^ could not reverse existing cell cycle events. PCSO-524^®^ (25 mg/kg) was fed to adult (four month old) *Atm*^−/−^ mice daily for 20 days. Immunostaining revealed that PCSO-524^®^ showed no significant effect in reversing neurons to exit previously initiated cell cycles. *** *p* < 0.001; one-way ANOVA with Dunnett’s multiple-comparison test; *n* = 4. Data are means ± SEM.

To test whether PCSO-524^®^ could protect primary neurons from CM, we pre-treated primary neuronal cultures for 2 h with PCSO-524^®^, before adding CM to a final concentration of 6.25%. In this situation, PCSO-524^®^ effectively prevented the appearance of neuronal cell cycle events (Figure 3G) and, at certain concentrations, was able to reduce the background of cycling cells by half. Despite this positive outcome, no concentration of PCSO-524^®^ prevented the cell death caused by CM (Figure 3H). To investigate whether the effect of PCSO-524^®^ was due to its anti-inflammatory actions, we repeated these experiments using CM from THP-1 cells that had been pretreatment with 16 µg/mL PCSO-524^®^ for 2 h before being exposed to Aβ. This modified conditioned medium, CM (PCSO), proved less neurotoxic since treatment of primary neurons with CM (PCSO) was less effective at the induction of cell cycle markers (Figure 4I) than the original CM, although this difference was not significant. Despite this more modest effect on the cell cycle phenotype, CM (PCSO) still led to a significant reduction in neuron number. Taken together, it appears that PCSO-524^®^ may offer double protection to both differentiated HT22 cells and primary neurons from cell cycle reentry.

### 2.4. PCSO-524^®^ Showed Protective Effect but Not Reversible Effect In Vivo

The data thus far showed that PCSO-524*^®^* has protective effects in both HT22 cells and neurons against in vitro challenges that mimic those of the Alzheimer’s disease brain. With these encouraging results, we performed an in vivo test of PCSO-524^®^ potency. Cell cycle-related neuronal death is the basis of our screening platform, and we took advantage of the fact that this form of neurodegeneration is found in other diseases in addition to Alzheimer’s [41]. The broader approach is particularly useful in the context of screening assays. While still useful for drug testing, experiments using Alzheimer’s disease-related models require many months to complete [16,42,43]. For efficiency, therefore, we have turned to the mouse model of ataxia telangiectasia (A-T) as our model of choice for exploring cell cycle-related neurodegeneration [44,45]. The cell cycle events in this model developed in a short, well-defined time window during the first postnatal month. In mice that are heterozygous and homozygous for Atm mutant alleles, Purkinje cells (the most seriously affected neurons in human A-T) express cell cycle proteins beginning between postnatal day 10 (P10) and P20 [3], making them an attractive model and a good choice for a first test in vivo. To test the efficacy of PCSO-524^®^, we used *Atm*^+/−^ and *Atm*^−/−^ mice (the *Atm^tm1Bal^* allele) from P10 to P30. PCSO-524^®^ was fed to the mice daily (25 mg/kg body weight) [46,47] from P10 to P30. Control animals were fed the same volume of the olive oil. After P30, mice were killed and processed as described in the Methods section.

We sectioned the immersed-fixed half brain in the sagittal plane and immunostained the 10 µm sections with the cell cycle markers PCNA and cyclin A2. As reported previously with a different *Atm* allele [*Atm^tm1Bal^*—see [4]], PCNA and cyclin A2 were ectopically expressed in the nuclei of Purkinje cells in untreated *Atm*^−/−^ and *Atm*^+/−^ mice; the Purkinje cells of wild type mice had only very low background staining (Figure 4A—vehicle). After PCSO-524^®^ treatment for 20 days, the expression of both PCNA and cyclin A2 was largely missing (Figure 4A—PCSO-524^®^, 4B,C). In addition to these effects in cerebellum, the cerebral cortex of *Atm*^−/−^ mice displayed a significant reduction in the levels of neuronal proteins compared with controls (Figure 4D). However, PCSO-524^®^ treatment successfully blocked the reduction of two neuronal markers: the nuclear protein, NeuN, and the synaptic vesicle protein, synapsin-I. Taken together our data demonstrated that if treatment is begun before the ectopic neuronal cell cycle events appear, PCSO-524^®^ can successfully block them in this A-T mouse model.

To investigate whether PCSO-524^®^ could reverse existing cell cycle events (a therapeutic effect), four months old *Atm*^−/−^ mice were fed with PCSO-524^®^ for 20 days (25 mg/kg body weight). Immunostaining of fixed brain sections showed that the percentage of PCNA-positive Purkinje cells was much higher at this stage than at P30 (Figure 4E). Unfortunately, PCSO-524^®^ treatment of adult mice proved unable to reverse the neuronal cell cycle phenotype (Figure 4E), indicating that the effect of PCSO-524^®^ in vivo is protective only.

## 3. Discussion

Unscheduled neuronal cell cycle activity is observed in populations of neurons that are lost as part of the pathogenesis of a variety of neurodegenerative conditions [6], including AD and A-T. This has led to the hypothesis that when mature neurons attempt to reenter a cell cycle, the result is cell death rather than division to produce two daughter cells [3,4,22,48]. Progressive loss of neurons is a major cause of neurodegeneration and explains the memory loss, brain shrinkage, and change of behavior that accompanies these conditions. The tight linkage between neuronal cell cycles and neuronal loss in AD, A-T, and other diseases has attracted the attention of many studies [3,4,5,6,9,48,49]. Coupled with evidence that such ectopic cell cycles directly drive the cell death process [11,12,50] and the relative ease with which this phenotype can be adopted as a screen, we have used this established biomarker as a screen for neurodegenerative disease. Previous studies have developed in vitro models mimicking neuronal cell cycle by neuroinflammatory stimulation [16,22,24]. The current study can be seen then as part of our effort to validate the use of this assay and cell cycle marker to screen compounds for their utility to protect neurons from cell cycle re-initiation. As illustrated by the results presented above, the approach has proven to offer a quick and feasible method to pre-screen drugs in vitro. Coupling this approach with the use of the *Atm^−^*^/−^ mouse model of neurodegenerative disease has allowed us to demonstrate that a commercially available marine natural product with strong anti-inflammation properties, PCSO-524^®^, has considerable potential as a preventive approach to treating neurodegenerative disease.

In our in vitro model, we have adopted a method reported previously by collecting the conditioned medium from Aβ stimulated THP-1 cells [25] and used it as a mitotic insult for both differentiated HT22 cells and primary neurons. While there are clear advantages to using an established cell line as a testing platform, there were also disadvantages that are apparent in our data. For both cells and neurons, Aβ stimulated THP-1 CM induced non-mitotic cells to re-enter a cell cycle (Figure 1D and Figure 3C). However, the effects of the CM on the two systems were not the same. CM did not reduce HT22 cell number, yet it significantly reduced neuronal cell number (Figure 3D). Yet, unexpectedly, the HT22 cells proved more sensitive than primary neurons to the toxic effect of high concentrations of PCSO-524^®^. In both cell types, however, PCSO-524^®^ was able to block CM from inducing cell cycle events in the differentiated cells. These differences stress the importance of using more than one cell type as a testing platform for any new compound.

The ability of PCSO-524^®^ to block the primary neurons from entering the cell cycle yet not block CM-induced cell loss is a curious finding. Two reasons for this dissociation of cell cycle and cell death present themselves. First, unlike HT22 cells, the primary neuronal cultures contain a wide variety of cell types—both neurons and non-neuronal cells. Non-neuronal cells, such as astrocytes, have been shown to be required to mediate the effects of neuroinflammation [51] in neuronal culture. The toxicity of CM, therefore, could be mediated by the non-neuronal cells in the culture. Second, and perhaps more likely, in addition to the pro-inflammatory cytokines, macrophages and microglia release a large amount of glutamate, nitrous oxide, and other small molecules as part of their inflammatory response (see discussion in Reference [52]). Compared to HT22 cells, primary neurons have more glutamate receptors [53], suggesting that in addition to a cytokine induced production of cell cycle proteins, the CM exerts part of its killing effects through the process of excitotoxicity, which is consistent with the previous report that glutamate appeared in the Aβ stimulated microglial conditioned medium [24]. This would mean that although PCSO-524^®^ can protect neurons from cell cycle reentry, it cannot block the acute cell death induced by CM.

Despite genetic and epidemiological evidence that neuroinflammation plays a substantial role in neurodegenerative diseases, such as AD [54,55,56], prospective human trials of nonsteroidal anti-inflammatory drugs (NSAIDs) have proven unsuccessful [56,57]. In AD mouse models, oral NSAIDs can prevent new neuronal cell cycle events but cannot reserve existing ones [42]. PCSO-524^®^ had a similar response in our in vivo model. If we administered PCSO-524^®^ before the advent of the cell cycle events in cerebellar Purkinje cells (i.e., before P10), we found that it offered a profound protective effect in both cortex and cerebellum. However, if we treated adult mice with exactly the same regimen, the protective effect was not apparent. The inability of PCSO-524^®^ treatment to reverse cell cycle events once they begin to appear suggests that, unlike initiation, the progression of AD may be independent of the presence of neuroinflammation. Thus, as with the therapeutic use of NSAIDs [57] and anti-amyloid therapy [58], as clinical trials are designed, great emphasis should be placed on beginning therapy as early as possible to prevent disease progression.

**Figure 5:**
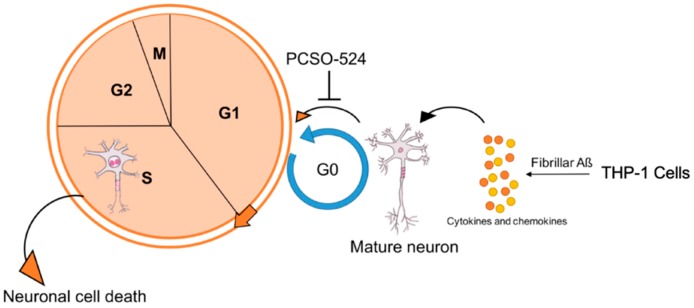
Diagram showing a proposed mechanism of PCSO-524^®^’s neuroprotective effect.

In summary, we have applied a quick and effective tool that used the abortive neuronal cell cycle events as a tool to screen compounds for their efficacy as neuroprotective agents. Using this method, we successfully identified PCSO-524^®^, the extract from New Zealand green-lipped mussel, as a strong candidate (Figure 5). The properties of PCSO-524^®^ suggest that it has considerable potential to stop or retard the initiation of neuronal cell cycle events and thus have utility in the treatment of neurodegenerative diseases such as AD.

## 4. Materials and Methods 

### 4.1. Animals

C57BL/6J-*Atm^−^*^/−^ mice (B6;129S4-Atmtm1Bal/J) were bred from stock originally obtained from The Jackson Laboratory (Bar Harbor, ME, USA). All mice used in this study were maintained in the Animal and Plant Care Facility of The Hong Kong University of Science and Technology (HKUST). Colonies were maintained by intercrossing heterozygous mice. All animal experiment protocols were approved by the Animal Ethics Committee at HKUST (protocol number #2016080), and animal care was provided in accordance with both institutional and Hong Kong guidelines that include government legislation and Hong Kong’s Code of Practice for Care and Use of Animals for Experimental Purposes, as well as International Guides and Codes of Practice on the Care and Use of Animals in Research.

To genotype mice from the Bal line, the following PCR primers were used:

PGK35:
5′-GGA AAA GCG CCT CCC CTA CCC-3′
Bal AT9:
5′-CCT CCT CAT ATT TGT AAC ACG CTG-3′
Bal AT12:
5′-TGT AAT GTG CCT TAA AGA ACC TGG-3′.

PCR was performed using the PCR ReadyMix Kit (E3004; Sigma-Aldrich, St. Louis, MO, USA). 

### 4.2. Drugs and Antibodies

PCSO-524^®^ was a generous gift from PharmaLink Company (Abu Dhabi, UAE). Antibodies against cyclin D1 (diluted 1:1000), cyclin A2 (diluted 1:1000), GAPDH (diluted 1:25,000), NMDAR (diluted 1:1000), and MAP2 (diluted 1:5000), were purchased from Abcam (Cambridge, MA, USA); PCNA (diluted 1:2000), antibody was purchased from Cell Signaling Technology (Danvers, MA, USA); Synapsin1 (diluted 1:20,000) and NeuN (diluted 1:3000) antibodies were purchased from Merck Millipore (Burlington, NJ, USA). Secondary antibody conjugated with fluorescent Alexa dye 488, 647 and Cy3 were purchased from Invitrogen (Carlsbad, CA, USA) and The Jackson Laboratory. Horseradish peroxidase (HRP)-conjugated secondary antibodies were purchased from Invitrogen.

### 4.3. Maintenance and Differentiation of HT22 Cells

HT22 cells were a generous gift from Professor Nancy IP. For the assays reported here, cells were seeded in 24-well plates (5000 cells per well) in Dulbecco’s modified Eagle’s medium (DMEM, Glibco, Carlsbad, CA, USA) supplemented with 10% fetal bovine serum (FBS, Life Technologies, Carlsbad, CA, USA) with 0.1% penicillin-streptomycin (Life Technologies) and cultured at 37 °C in a humidified atmosphere of 5% CO_2_. HT22 cells were differentiated in DMEM with 1mM dbcAMP (Sigma) for six days [37].

### 4.4. Embryonic Cortical Neuron Culture

The culture surface of each well was coated with poly-l-lycine (0.5 mg/mL, diluted in borate buffer, Sigma). Tissue for cortical neuron culture was harvested from the cerebral cortex of E16.5 embryos. On E16.5, the gravid female was killed, the embryos removed, and the cerebral cortices isolated and dissected into phosphate buffer saline (PBS) with 1 mg/ml glucose (Sigma). The cortical tissue was cut into small pieces (2–3 mm^3^) and treated with 0.25% trypsin-EDTA (Sigma) for 10 min at 37 °C. Tissues were then washed in DMEM with 10% FBS to inactivate the trypsin, followed by washing in Neurobasal medium (Life Technologies). The trypsin digested tissue was then triturated to produce a single cell suspension. Cells were plated onto the poly-l-lysine coated plates at 48,000 cells/well in Neurobasal medium supplemented with B27 (Life Technologies), 2 mM glutamax (Life Technologies), 100 U/mL penicillin (Life Technologies), and 100 μg/mL streptomycin (Life Technologies). Cultures were maintained at 37 °C in a humidified 5% CO_2_.

### 4.5. THP-1 Cell Culture and Conditioned Medium

Human THP-1 cells (an immortalized human macrophage line) were grown in RPMI 1640 (Life Technologies) with 10% heat inactivated FBS, 5 × 10^−5^ M β-mercaptoethanol (Sigma), and 0.1% penicillin-streptomycin (Life Technologies) at 37 °C in 5% CO_2_. We followed a previous report in stimulating the THP-1 cells [22], where 2.27 μL of 220 µM fibrillar Aβ1-42 was added to sterile 24-well plates and allowed to air dry. The THP-1 cells were then seeded on this substrate (100,000/well) and cultured for 72 h in 500 μL DMEM with 10% FBS for HT22 cell assays, or with serum-free Neurobasal medium for neuronal assays. After the 72 h incubation, the conditioned medium was used immediately.

### 4.6. Histochemistry and Immunocytochemistry

To detect DNA synthesis, 10 μM 5-ethynyl-2′-deoxyuridine (EdU, Life Technologies) was added to the culture medium 2 h before fixation in 4% paraformaldehyde (Sigma) in PBS for 20 min at room temperature [59]. Cells were then processed for immunocytochemistry. After washing with PBS with 3% bovine serum albumin (Sigma), cells were permeabilized with 0.5% Triton-X100 in PBS and stained with Click-iT chemistry (Life Technologies).

For immunohistochemistry, non-specific binding sites were blocked by a 1 h incubation with 5% donkey serum (Sigma) in PBST (PBS with 0.3% TritionX-100). Cells were incubated overnight at 4 °C with primary antibody at the dilution in blocking solution, washed in PBS and incubated with secondary antibody diluted 1:500 in blocking solution for 1 h at room temperature. DAPI was applied as a nuclear counterstain before the cultures were mounted in Hydromount and analyzed on a fluorescence microscope (Olympus DP80).

For quantification, five views per coverslip were randomly chosen based on DAPI staining. The cell number of differentiated HT22 cells was counted based on the number of NMDAR positive cells with differentiate morphology. Neuronal number was counted based on MAP2-positive cells. EdU, cyclin D1 positive cells was counted based on positive staining signals in the nucleus. 

For analysis of in vivo cell cycle events, mice were anesthetized with Avertin (tribromoethanol, 250 mg/kg) and perfused transcardially with PBS. One hemibrain was snap-frozen and stored at −80 °C. The other hemibrain was immersion-fixed in 4% paraformaldehyde in PBS for a minimum of 24 h. The fixed brain was then embedded in OCT, quickly frozen in powdered dry ice, cryostat sectioned at 10 µm (Thermo CryoStar NX70 Cryostat), and mounted on glass slides. After air drying, the slides were stored at −80 °C until use. For immunostaining, the thawed slides were subjected to antigen retrieval with a citrate buffer (pH 6.0, 95 °C, 10 min). The sections were then blocked with 5% donkey serum in PBST at room temperature. After rinsing, primary antibody was added and incubated overnight at 4 °C. The sections were then rinsed and incubated with Alexa Fluor-conjugated secondary antibody for 1 hour at RT. Nuclei were counterstained with DAPI. 

### 4.7. Western Blot of Cultured Cell Lysates

Medium was aspirated and cells were lysed in radioimmunoprecipitation assay buffer (RIPA buffer, Merck Millipore) supplemented with EDTA-free protease inhibitor and phosphor-stop cocktail (Roche, 1 tablet per 10 mL RIPA extraction buffer). Lysates (10 µg protein) were subjected to sodium dodecyl sulfate polyacrylamide gel electrophoresis (SDS-PAGE). The gel-separated proteins were then transferred to PVDF membrane (Bio-Rad, Hercules, CA, USA). The membranes were blocked in TBS containing 0.1% Tween-20 and 5% skimmed milk. The blots were rinsed, primary antibodies applied and incubated overnight in room temperature. After rinsing, horseradish peroxidase-conjugated secondary antibodies were added for 1 hour in room temperature followed by a final rinse. Antibody-stained bands were visualized with luminol-based enhanced chemiluminescence HRP substrate (Bio-RAD). The intensity of bands was quantified utilizing ImageJ.

### 4.8. ELISA

The concentration of pro-inflammatory cytokines TNFα and IL1β in the conditioned medium were qualified by the human TNFα and IL1β ELISA kit from R&D systems.

### 4.9. JC1 Dye Incorporation

JC1 dye (Life Technologies, 1 µg/mL) was incubated with primary neurons for 20 min in 37 °C. Cells were then washed twice with Neurobasal medium and once with PBS. The dye fluorescence intensity was measured by EnVision multilabel reader (EnVision Xcite, Perkin Elmer, Waltham, MA, USA) at 490 nm excitation and 535 nm emission (for JC1 monomers) and at 595 nm (for JC1 aggregates). The ratio of monomer to aggregate (535/595) was calculated, the higher value representing the polarization of the mitochondrial membrane.

### 4.10. Drug Treatment In Vivo

The PCSO-524^®^ solution was freshly prepared every three days. The stock solution was dissolved in olive oil to 50 mg/mL and fed to the animals such that each received a dose of 25 mg/kg. Each animal was manually restrained by holding the skin on the back of the neck with thumb and forefinger and anchoring the tail between the little finger and the palm. The end of a plastic micropipette tip containing the PCSO-524^®^ solution was placed on the mouth while the solution was expelled slowly, taking care to leave the nares free for breathing.

### 4.11. Statistical Analysis

Data are expressed as mean ± SEM (standard error of the mean) for each group. The statistical significance of changes in the different groups was evaluated by one-way ANOVA using GraphPad Prism software (Version 7, GraphPad Software, San Diego, CA, USA). Statistical significance was set such that *p* ≤ 0.05 was considered as significant.
